# The Academy of Medicine Paris

**Published:** 1848-09

**Authors:** 


*The Academy of Medicine, Paris,* has named a commission charged with the duty of drawing up a work on all the questions which attach themselves to the process of poisoning by the salts of lead, and in particular on the prophylactic means to be applied to establishments where these products are fabricated. This subject involves a crowd of questions in therapeutics, in chemistry, and in public hygiene of the highest interest, and which will be worthy of the profoundest inquiry. Such are, for instance, the question whether the solubility of the salts is or is not a condition indispensable to their absorption—the question of chemical neutralizing agents. The present great development of the poisoning diathesis gives importance to any extension of our means of combating its awful results. We shall look with interest for the report of the academy, to lay it before our readers.—*Ibid.*

